# Monument to Dr. Horace Wells

**Published:** 1870-04

**Authors:** 


### MONUMENT TO DR. HORACE WELLS.

At a regular meeting of the Hartford Society of Dentists, held at the office of Dr. C. M. Hooker, April 11, 1870, the following resolutions, presented by Dr. McManus, were adopted:

*Resolved*, That the natural gratitude due to the memory of public benefactors, imperatively demands that the city of Hartford and the State of Connecticut, with the medical and Dental professions, cause a suitable monument to be erected in the public park of this city, in memory of Dr. Horace Wells, the discoverer of anæsthesia.

*Resolved*, That Drs. J. M. Riggs, James McManus, E. E. Crofoot, C. M. Hooker and Wm. Blatchley, be and are hereby appointed a committee, whose duty it shall be to cause suitable petitions to be presented to the City Council of Hartford and the General Assembly of this State in furtherance of this object, and to obtain signatures to the same.

In connection with the above, we copy the following from a Hartford paper.—Ed. Reg.

### THE DISCOVERER OF ANÆSTHESIA.

The Dental Association of this city have passed resolutions (which we copy) awarding to Dr. Horace Wells the merit of the discovery of anæsthesia; and it is proposed to erect in this city a suitable monument or statue to his memory, and in honor of the great discovery which has brought such blessings to the world. We are gratified to witness this interest on the part of the Dental Association. To Dr. Wells belongs the honor of the discovery. The proof is so conclusive that no honorable and unbiased man can ignore it. We recently laid before our readers the main facts in the case. Even Prof. Simpson, of the University of Edinburg, in a late paper upon this subject awards to Dr. Wells the merit of "extracting teeth from a dozen or more patients, rendered insensible by inhaling nitrous oxide gas, at least two years previous to the time when Dr. Jackson and Dr. Morton resorted to its use. We trust there is a sense of justice in Connecticut and in Hartford city, which shall demand and secure all that the Dental Association claim shall be done.

